# “StudiCare Mindfulness”: effects of an online mindfulness-based intervention on mindfulness, stress, and interoception in university students – a randomized controlled trial

**DOI:** 10.1186/s40359-026-04631-1

**Published:** 2026-04-29

**Authors:** Christine Marie Schillings, Dana Schultchen, Ann-Marie Küchler, Matthias Mack, Harald Baumeister, Olga Pollatos

**Affiliations:** 1 Department of Clinical and Health Psychology, Institute of Psychology and Education, Ulm University, Ulm, Germany; 2 Impact and Prevention of Mental Disorders, Institut de Recerca Sant Joan de Déu (IRSJD), Esplugues de Llobregat, Spain; 3 Department of Clinical Psychology and Psychotherapy, Institute of Psychology and Education, Ulm University, Ulm, Germany; 4 Department of Clinical and Biological Psychology, Institute of Psychology and Education, Ulm University, Ulm, Germany; 5 German Center for Mental Health (DZPG), Partner-Site Mannheim- Heidelberg-Ulm, Mannheim, Germany; 6 German Center for Child and Adolescent Health (DZKJ), Partner-Site Ulm, Ulm, Germany

**Keywords:** Mindfulness, Mindfulness-based intervention, Online intervention, Stress, Interoception, Hair cortisol, University students, Randomized controlled trial

## Abstract

**Background:**

University students experience elevated stress levels that increase the risk for developing mental disorders. Online mindfulness-based interventions (MBIs) represent a promising low-threshold approach to improve health-related variables. However, evidence regarding their effects on psychobiological stress markers and interoceptive abilities remains limited.

**Methods:**

This randomized controlled trial investigated the efficacy of an 8-week guided online MBI (StudiCare Mindfulness) in university students (*n* = 60) compared to a waitlist control group (*n* = 61). Assessments were conducted at baseline (T0), after eight weeks (T1), and 6-month follow-up (T2). Primary outcome was mindfulness (Freiburg Mindfulness Inventory). Secondary outcomes included perceived stress (Perceived Stress Scale-4), psychobiological stress (hair cortisol), interoceptive accuracy (heartbeat perception task), and interoceptive sensibility (confidence ratings, Body Perception Questionnaire). Hierarchical linear models were employed for data analysis.

**Results:**

Significant time × group interactions demonstrated that the intervention group significantly improved in mindfulness (*β* = 3.137, *p* = .002) and showed reductions in perceived stress (*β* = − 0.989, *p* < .001) compared to controls, with effects maintained at follow-up. No significant intervention effects were observed for psychobiological stress and interoceptive abilities.

**Conclusions:**

The guided online MBI effectively enhanced mindfulness and reduced perceived stress in university students, with sustained effects at 6-month follow-up. However, the intervention did not significantly affect psychobiological stress markers or interoceptive processing, suggesting differential sensitivity of psychological versus psychobiological stress dimensions to online MBIs. At the same time, the absence of effects on psychobiological measures should be interpreted with caution, as these outcomes may have been subject to additional uncontrolled sources of variability.

**Trial registration:**

German Clinical Studies Trial Register TRN: DRKS00014701, trial registration date: 07.05.2018.

## Background

Current evidence demonstrates that university students exhibit significantly elevated stress levels compared to general population norms [1–4], with some evidence suggesting elevated rates of stress also relative to age-matched non-student peers [5]. Potential stress factors students are regularly exposed to include academic workload and pressure, time management, and financial concerns [6, 7]. Significantly, high stress levels are a critical risk factor in the development of mental disorders such as depressive or anxiety disorders [2, 8]. An epidemiological study based on data of 72.288 first-year students across 77 universities worldwide reveals that 67% have a lifetime history of at least one mental disorder, with 31% reporting symptoms in the previous 12 months [9].

Preventing elevated stress levels is essential to reduce the risk of university students to develop mental health disorders. Previous research has shown that, despite the availability of free health and counselling services at most universities, many students do not seek professional help [10]. Thereby, reported barriers to accessing these services include limited time, a preference to address issues on their own, insufficient knowledge of access to professional treatment, and a perceived lack of need for professional help [10].

In this context, online- and mobile-based psychological interventions (IMIs) have been identified as a promising approach to overcome these barriers, providing several advantages, such as low-threshold access, reducing stigma, enhancing cost-effectiveness, and providing greater flexibility in terms of time and location of use [11–15]. A growing body of evidence supports the efficacy of online interventions, suggesting they can be as effective as traditional face-to-face programs for both clinical and non-clinical populations [16–20]. Further research [21–24] has shown high levels of acceptance and usability of online- and mobile-based interventions among university students. An increasing number of studies also indicate that online interventions can effectively improve mental health outcomes such as depressive or anxiety symptoms and stress [25–28]. There is evidence that guided IMIs are superior to unguided interventions with respect to the effectiveness in reducing symptoms of diverse mental disorders [29–33].

One approach to cope with stress and mental disorders are mindfulness-based interventions (MBI’s). Mindfulness has been conceptualized as a state of being aware and focused on the present moment in an open, accepting and non-judgmental way [34–37]. Mindfulness-based interventions are theorized to reduce stress through several mechanisms, including enhanced attentional control, body awareness, and improved emotion regulation [38]. Numerous studies [39–45] and meta-analyses [29, 31, 32, 46, 47] have shown positive effects of online MBI on self-reported health outcomes such as stress, depression, and mindfulness. In particular, a recent meta-analysis examining online MBI’s for university students [46)] reported standardised mean differences of 0.71 (95% CI 0.15 to 1.25) for mindfulness and −0.58 (95% CI −0.79 to −0.37) for stress. Furthermore, in a study examining the effectiveness of a guided IMI “StudiCare Mindfulness” designed for university students [48], enhanced mindfulness (*d* = 1.37, 95% CI 1.01 to 1.73) and reduced perceived stress levels (*d* = − 0.92, 95% CI −1.25 to −0.58) were found compared with a waitlist control group. Similarly, the results of the follow-up study of Küchler et al. [41] comparing a guidance on demand and an unguided version of the same intervention with a waitlist control group, showed improved mindfulness (*d* = 0.91 to 1.06, 95% CI 0.66 to 1.32) and decreased perceived stress (*d* = –0.32 to −0.63, 95% CI –0.88 to −0.07) in both intervention groups, with effects maintained at 6-month follow-up. However, most of these studies are based on self-reported outcomes. Thus, psychophysiological measures are needed to complement self-report data [49], in particular in IMI research, to provide a more comprehensive understanding, convergent evidence, and stronger validity for psychological constructs such as stress. Therefore, the present study is based on the guided version of IMI “StudiCare Mindfulness” [41, 48], complemented by psychophysiological outcomes.

There is profound evidence that identified hair cortisol is an appropriate psychobiological biomarker to assess chronic stress, reflecting stress exposure over weeks or months, and less influenced by acute stressors or circadian rhythms [50–53]. Previous studies investigating the effects of MBI on hair cortisol are limited and showed mixed findings. In a sample of academic staff, reduced hair cortisol and perceived stress levels were found due to an 8-week offline mindfulness intervention compared to a waitlist control group [54]. Remarkably, the small sample size of this study (*N* = 30) needs to be considered. In contrast, for an 8-week body scan intervention, decreased hair cortisol levels were found compared to an active control group [55]. However, a recent study [56] found that hair cortisol levels in a student sample did not show the expected decrease after either an online 4-week mindful stretching intervention with mindful breathing or an online mindful stretching only condition, whereas self-reported mindfulness increased in both groups. Similarly, no reduction in hair cortisol levels was found for an 8-week online-based Acceptance and Commitment Therapy intervention compared to a face-to-face mindfulness training for students [57]. Conclusively, further research on the long-term effects of online MBI on psychobiological stress in students is needed.

A construct closely linked to stress processing is interoception [58, 59], which is defined as the conscious or unconscious perception and processing of internal bodily signals such as cardiovascular, gastrointestinal or respiratory signals [60]. Interoception is conceptualized as a multidimensional construct encompassing interoceptive accuracy (the objective ability to detect internal bodily signals), interoceptive sensibility (subjective beliefs about one’s own ability to perceive internal bodily signals), and interoceptive awareness (the metacognitive correspondence between accuracy and sensibility) [61]. Several theoretical frameworks (e.g. [58, 59, 62, 63], propose that interoception plays a central role in stress and emotion processing. In particular, high interoceptive abilities are associated with a better perception as well as a more intense emotion experience and better emotion regulation [64–67]. Conversely, impaired interoception has been found in populations with long-term stress [68] and mental disorders such as depression [69], suggesting a close link between interoceptive deficits and stress-related psychopathology.

Mindfulness-based interventions, with their emphasis on present-moment body awareness, are theorized to enhance interoceptive processing by training sustained, non-judgmental attention to bodily sensations [38, 70, 71]. However, empirical evidence for this pathway remains limited. Studies investigating the effects of offline mindfulness interventions on interoception [72–75] and a chatbot-based intervention fostering mindfulness, stress, and interoception [76] have shown mixed results. So far, research on the effects of online MBI on interoceptive abilities is particularly sparse: Only one study [77] has investigated effects on interoception, measuring only interoceptive sensibility and showing improvements in some subscales of the Multidimensional Assessment of Interoceptive Awareness [78, 79].

In sum, to the best of our knowledge, no previous study has examined the effects of an online MBI on mindfulness, interoception, and both perceived and psychobiological stress. In order to close the gaps in research, the present two-armed randomized controlled trial aims to investigate the effects of an 8-week guided online MBI for college students “StudiCare Mindfulness”, on mindfulness, interoception, perceived and psychobiological stress compared to a waitlist control group. The following hypotheses were derived:

1. Mindfulness (primary outcome), as measured via the Freiburg Mindfulness Inventory (FMI; [80]), increases in the intervention group after the online MBI compared to the waitlist control group.
2. Stress in the intervention group decreases after the online MBI compared to the waitlist control group.
  - Perceived stress, as assessed via the Short Form Perceived Stress Scale (PSS-4; [81]) in the intervention group decreases after the online MBI compared to the waitlist control group.
  - Psychobiological stress, as assessed via hair cortisol, decreases in the intervention group after the online MBI compared to the waitlist control group.
3. Interoception in the intervention group increases after the online MBI compared to the waitlist control group. More specifically, an increase for interoceptive accuracy (a) assessed via the heartbeat perception task [82] and (b) interoceptive sensibility (IS) as assessed via confidence ratings is expected.

## Methods

### Participants

Participants were recruited by flyer, social media, and word-of-mouth advertisement as well as e-mail announcements. An online screening questionnaire was used to investigate if the participants fulfilled the following inclusion criteria: (a) age of 18 or above, (b) enrolled at a university or college in Ulm (as the online intervention is part of a nationwide study and in-person availability was important for collecting the hair samples), (c) sufficient knowledge of the German language, (d) internet access, (e) low to moderate mindfulness (FMI < 37). The mindfulness criterion was applied to target individuals with the greatest potential benefit from mindfulness training, consistent with previous studies [41, 48], which had used the same cut-off and corresponding to FMI mean values in the general population [80]. Students currently undertaking a psychotherapy or any kind of mindfulness intervention were excluded from the study. Participants were informed that “StudiCare Mindfulness” cannot replace psychotherapy and were recommended to seek counselling in case of distinctive mental health problems. Additionally, they were offered information on alternative treatment options and contact details (this information was also given to participants not meeting the selection criteria).

The study was approved by the Ethics Committee of Ulm University and all procedures were conducted in accordance with the Declaration of Helsinki. Prior to taking part in the study, all participants had read and signed the informed consent. The trial was designed, conducted, and reported in concordance with the CONSORT 2010 guidelines [83]. Moreover, the trial had been registered prior to the recruitment at the WHO International Clinical Trials Registry Platform via the German Clinical Studies Trial Register (DRKS; ID: DRKS00014701). Data collection was pseudonymised and only authorised study personnel obliged to secrecy got access to the data. After the participants had successfully completed all parts of the study, they received a monetary compensation of five euros for their participation in the follow-up-measurement. Importantly, participation in the online intervention was used as the main incentive.

### Procedure and materials

**Fig. 1:**
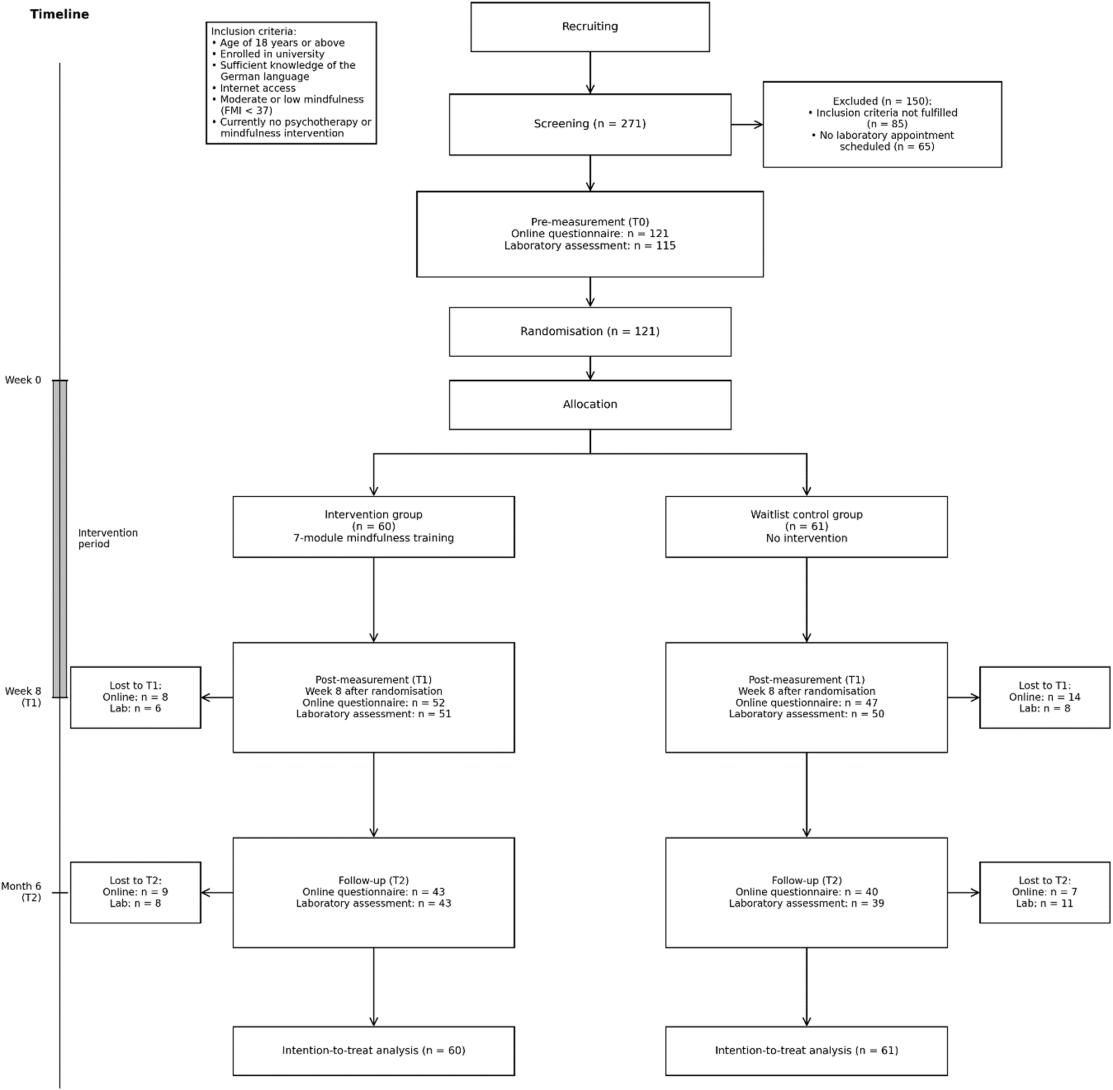
Flow chart of the study procedure

Figure 1 illustrates the participant flow throughout the study, including sample sizes at each assessment point. After passing the screening questionnaire, students were allocated based on their college affiliation either to the present trial (students from Ulm University and other Ulm colleges) or to a partner trial (all other colleges). A total of *N* = 121 students were randomly assigned to either the intervention group or the waitlist control group. The detailed design of the present two-armed, parallel randomized controlled trial is described in the study protocol [84]. Participants included via the screening were tested in the laboratory at three measurement points (T0: at the beginning; T1: eight weeks after randomisation and T2: six months after randomisation), where all outcomes were assessed. Assessments took place in a laboratory of Ulm University. When participants came to the laboratory for the first assessment, they got the opportunity to again read the information about the purpose of the study and the procedure they had received as an online version before. Afterwards, they had to sign an informed consent to take part in the study. Before the participants conducted the heartbeat perception task [82], they underwent a five-minute baseline assessment of heartrate, electrodermal activity, and respiration via a BIOPAC MP150. Additionally, the participants’ weight and height were measured. The testing procedure was identical across all three measurements, apart from height measurement, which was only assessed at T0. After each laboratory assessment, the participants completed an online questionnaire (www.unipark.de) in which they provided demographics (e.g., age, gender, subject) and completed a battery of psychological questionnaires including FMI, Body Perception Questionnaire, and PSS-4. After the participants had finished the pretest, they were randomly allocated to the intervention group (*n* = 60) or the waitlist control group (*n* = 61) by an independent researcher not otherwise involved and therefore blinded to all processes of the study. Via an automated, online-based randomisation program (www.sealedenvelope.com), permuted block randomisation was performed with an allocation ratio of 1:1 and variable block sizes of 2 and 4 (randomly arranged).

### Outcome assessment

#### Primary outcome mindfulness

Mindfulness was assessed by the 14-item short scale of the Freiburg Mindfulness Inventory (FMI [80]), consisting of a 4-point Likert scale, which ranges from 1 (= rarely) to 4 (= almost always). A sum score was calculated, with higher scores indicating higher mindfulness. Internal consistency of the FMI ranged from α = 0.60 to 0.83 across measurement points, which is comparable to values reported in similar student samples (α = 0.73 [48]; α = 0.84 [85]).

#### Secondary outcomes

##### Perceived stress

The participants’ acute perceived stress as the degree to which situations in one’s life are rated as stressful was measured by the German version of the Perceived Stress Short Scale (PSS-4) [81]. The scale ranges from 0 (= never) to 4 (= very often). Results are calculated as sum scores, with higher scores indicating higher perceived stress. Internal consistency of the PSS-4 ranged from α = 0.70 to 0.79 across measurement points, which is in line with psychometric properties reported in previous studies based on student samples (α = 0.77 [86]; α = 0.77 [85]).

##### Interoceptive accuracy

Interoceptive accuracy (IAc) was assessed using the heartbeat perception task (82). Thereby, the participants were instructed to focus on their own heartbeats and to count them silently during four different intervals (lengths: 25, 35, 45, and 60 s), without getting any information about the lengths of the intervals or any feedback about their performance quality. Moreover, they were briefed to sit in a relaxed position to avoid movements and not to use manipulating strategies like taking their pulse or stop breathing. If preferred, they could close their eyes during the task. For every interval, the investigator gave the participants a start and a stop signal. Immediately after every stop signal, the participants had to report their counted heartbeats and their confidence ratings (see interoceptive sensibility). Before the first interval started, the participants had to perform a 10-second training interval. For the heart rate recording, BIOPAC MP150 with a sampling rate of 1000 Hz was used. Analyses of cardiovascular signals were conducted via the Software Acknowledge (version 4.4). The averaged heartbeat perception scores were calculated using the following equation:

$$\begin{aligned}&IAc\text{} Score\mathrm{=}\frac{1}{4}\text{}\sum\:\\&(1\mathrm{-}\frac{\left(\left|recorded\text{} heartbeats\mathrm{-}counted\text{}heartbeats\right|\right)}{recorded\text{}heartbeats}\end{aligned}$$

The score ranges from 0 to 1; higher scores indicate higher IAc representing a higher sensitivity for cardiovascular signals [87].

#### Interoceptive sensibility

Interoceptive sensibility (IS) was assessed using two measures: confidence ratings and the Body Perception Questionnaire (BPQ [88]). Following each interval of the heartbeat perception task [82], participants rated their confidence in the accuracy of their counted heartbeats on a scale from 0 (= total guess/no heartbeat perception) to 10 (= complete confidence/full perception of heartbeats). Additionally, the awareness subscale of the BPQ was assessed, which comprises 45 items assessing bodily signal awareness on a 5-point Likert scale (1 = never; 5 = always). Raw scores were converted to standardised t scores (*M* = 50, *SD* = 10). Internal consistency of the BPQ ranged from α = 0.93 to 0.96 across measurement points, indicating excellent reliability consistent with previous findings in a college sample [89].

### Intervention: online mindfulness-based intervention vs. waitlist control group

StudiCare Mindfulness is an enhanced version of a previously evaluated IMI [41, 48] expanded from the original intervention through the addition of two core modules and two booster modules. Therefore, the revised program consists of seven sequential core modules (45–60 min each, completed weekly) and two booster modules that are unlocked at four- and twelve-weeks post-intervention to support sustained treatment effects. The intervention combined elements from Mindfulness-Based Stress Reduction (MBSR; [35]), body-focused practices, Acceptance and Commitment Therapy (ACT [90]) , and stress management techniques [91].

The intervention consisted of seven target-group-specific modules taking approximately 50 to 60 min each (see Table 1). Four and twelve weeks after the participants had completed the seventh module, two shorter booster sessions were activated in order to ensure long-time effectiveness. The contents were presented via texts, images and interactive elements such as weekly homework assignments, downloadable materials including handouts, audio files, and a mindfulness diary.

The intervention was available on the platform “Minddistrict” (www.minddistrict.com*)*, a company specialised in the provision of internet-based health interventions. Participants got access to the platform via their personal username and password on a 24/7 basis. All transferred data were secured based on ISO27001 and guidelines NEN7510. Participants of the intervention group received support by trained and supervised e-coaches. The e-coaches gave semi-standardised feedback after participants had finished their modules within two working days, following an e-coach manual. The participants had the possibility to contact their e-coach in case of occurring questions. In addition, the intervention group had access to treatment as usual, i.e., participants could use other support or treatment options which was monitored in order to control for potential confounding effects.

The waitlist control group received an information leaflet informing them about alternative support options such as university counselling services, psychotherapy, or helplines as well as the advice to seek help in case their well-being was decreasing. They had unrestricted access to any usual treatment options. Six months after randomisation, participants of the control condition got access to an unguided version of the intervention.

**Table 1:** Overview of the different topics and contents of each module

Module	Aims and content	Examples of exercises and assignments
1. Being in the here and now	Introducing the concept of mindfulness	Reviewing most and least mindful moments of the day; practicing Body Scan; taking a mindful walk
2. Mindful body perception	Practicing awareness of body signals	Testing one’s heartbeat perception; practicing “heart meditation”; mindful eating and drinking
3. A new perspective on stress	Distancing oneself from stress-inducing thoughts	Identifying former ways of coping with stress; learning techniques to challenge automatic thoughts; meditation exercise
4. Developing beneficial thoughts	Getting to know alternative ways of thinking	Identifying one’s “stress patterns” and developing and internalising beneficial thoughts; practicing a breathing meditation
5. What makes your life valuable?	Identifying one’s values and pursuing one’s goals	Writing a speech for one’s own 70th birthday; setting and pursuing goals with the SMART technique; meditation exercise
6. Being mindful towards yourself	Learning how to appreciatively accept one’s personality traits	Exercise to identify different personality traits and corresponding automatic reactions; learning to accept and appreciate all personality traits
7. Training your body and senses	Exercising the ability to enjoy and getting acquainted with the practice of yoga	Mindful chocolate eating exercise; mindful yoga exercises
Booster-Session 1 (after 4 weeks)	Repeating module 1 to 3 and mindfulness exercises	Choosing favorite mindfulness exercises; setting goals for their implementation in the coming weeks
Booster- Session 2 (after 12 weeks)	Repeating modules 4 to 7 and ensuring long-term integration of mindfulness into daily life	Reviewing pursuit of goals in the last two months; identifying potential barriers and developing solutions

#### Psychobiological stress: hair cortisol

Hair samples (2 cm in length) were collected from the posterior vertex, a position with relatively steady growth rates [92, 93]. Assuming an average growth rate of approximately 1 cm/month, this segment reflects integrated cortisol secretion over the preceding two months [93, 94]. The hair samples were placed in a microcentrifuge tube. Samples were washed three times with 1 mL isopropanol to remove external contaminants, and the solvent was allowed to evaporate overnight. Three stainless steel beads (5 mm) were then added to each tube, and hair was pulverised using a TissueLyser LT (Qiagen, Germany) for 10 min at 60 Hz. Subsequently, 1 mL methanol was added, and the sample was pulverised again for 10 min at 60 Hz. After inversion of the tubes, another shaking step at 50 Hz was performed to ensure homogenous extraction. The methanol extraction continued with incubation on an orbital shaker for 20 h, followed by centrifugation at 600 × *g* for 10 min. A 600 µL aliquot of the supernatant was transferred to a new tube, and the methanol was evaporated to dryness using a SpeedVac concentrator. The dried extracts were stored at 80 °C. For the assay, samples were reconstituted in 150 µL of assay buffer and analysed according to kit instructions.

Cortisol concentrations were determined using a commercial competitive ELISA kit (DetectX^®^ Cortisol, Arbor Assays, USA) following the manufacturer’s protocol. Briefly, 50 µL of samples or standards were added in duplicate to the wells of a microtiter plate. For non-specific binding (NSB) wells, 75 µL of 1× Assay Buffer was added, and for the zero standard (B0) wells, 50 µL of 1× Assay Buffer was added. Subsequently, 25 µL of DetectX^®^ Cortisol Conjugate and 25 µL of DetectX^®^ Cortisol Antibody were added to each well (except NSB wells). The plate was sealed and shaken at room temperature (700–900 rpm) for 1 h. Following incubation, the plate was washed four times with 300 µL of 1× Wash Buffer per well and blotted dry. Then, 100 µL of TMB substrate was added to each well and the plate was incubated for 30 min at room temperature in the dark without shaking. The reaction was stopped by adding 50 µL of Stop Solution to each well, resulting in a color change from blue to yellow. Optical density (OD) was measured at 450 nm within 10 min using a microplate reader. Cortisol concentrations were calculated using a four-parameter logistic (4PL) standard curve. The mean OD values of duplicates were corrected by subtracting the NSB mean OD. For each sample, the net OD was expressed as a percentage of the zero standard (%B/B0). Cortisol concentrations (pg/mL) were interpolated from the 4PL regression of the standard curve, and results were adjusted for any dilution factor applied during sample preparation.

#### Intervention adherence

Intervention adherence was operationalized as the number of completed core modules and booster sessions (range: 0–9) among participants in the intervention group. A module was considered completed when all exercises and contents had been finished.

### Data analysis

All analyses were conducted according to the intention-to-treat principle. Descriptive statistics, including means, standard deviations, frequencies, and percentages were calculated for demographic data and all outcome variables. To account for the nested longitudinal data structure, hierarchical linear models were calculated to investigate the intervention effects across three measurement points: pre-assessment (T0), post- assessment (T1), and follow-up (T2). The measurement points (level 1) were nested within the participants (level 2). The regression analyses included the three measurement points. Statistical analyses were conducted using the software R, in particular, the packages lme4 [95], lmerTest [96], r2mlm [97], and robustlmm [98]. The predictor variable time had an interpretable zero point and the dichotomous predictor group was dummy-coded. Due to assumed interindividual and intraindividual differences, random intercept and random slopes models were calculated, where possible. Otherwise, random intercept and fixed slope models were calculated. Parameters were estimated using Restricted Maximum Likelihood (REML), to minimise bias [99]. The statistical significance level for all analyses was set at *p* ≤ .05.

For analysing psychobiological stress, cortisol samples were integrated if they were within range of the standard curve and had a coefficient of variation (CV) < 20% between the investigated duplicates of one sample. The CV cut-off value is based on recommendations [100]. Given considerable dropout in psychobiological stress measures at T2, we restricted our analyses to changes between T0 and T1. The data analysis of psychobiological stress was conducted using the *R* package robustlmm [98] to reduce the influence of potential outliers through robust estimation methods. In addition to the pre-specified hierarchical linear models, we conducted exploratory bivariate correlations between perceived stress and psychobiological stress across the available measurement time points. These analyses were not pre-registered and were motivated post hoc by previous findings indicating that psychologicaland psychobiological indicators of stress are often only weakly associated [55].

## Results

### Participants

The final sample included *n* = 60 participants (90.2% female) in the intervention group and *n* = 61 (86.6% female) in the control group. The demographic data and the relevant descriptive statistics per group are depicted in Table 2. There were no significant differences between the groups except for BMI.

**Table 2:** Sociodemographic and relevant descriptive statistics of the sample at baseline by group

Variable		IG (*n* = 60)	CG (*n* = 61)	Test statistic	*p*
		*n* (%)	*n* (%)	χ²(1)	
Gender	Female	52 (86.7)	55 (90.2)	0.10	0.751
	Male	8 (13.3)	6 (9.8)		
German native speaker	Yes	59 (98.3)	58 (95.1)		
	No	1 (1.7)	3 (4.9)		
Marital status	Single	41 (68.3)	47 (77.0)		
	In a relationship	18 (30.0)	12 (19.7)		
	Married	1 (1.7)	2 (3.3)		
Student status	Full-time	59 (98.3)	58 (95.1)		
	Part-time	0 (0.0)	2 (3.3)		
	Other	1 (1.7)	1 (1.6)		
Field of study	Psychology	46 (76.7)	43 (70.5)		
	Medicine	8 (13.3)	11 (18.0)		
	Economics	2 (3.3)	3 (4.9)		
	Other	4 (6.7)	4 (6.6)		
		*M* (*SD*)	*M* (*SD*)		
University semesters		5.30 (4.12)	5.39 (4.73)		
				*t*(119)	*p*
Age		22.38 (3.20)	21.80 (2.95)	−1.04	0.301
Mindfulness (FMI)		31.60 (4.92)	31.44 (4.28)	−0.02	0.851
IS (BPQ)		2.77 (0.73)	2.78 (0.71)	0.03	0.973
Perceived stress (PSS)		7.33 (3.32)	6.67 (2.34)	−1.27	0.207
				*t*(113)	*p*
BMI		21.85 (3.79)	23.27 (3.32)	2.15	0.034
IAc		0.57 (0.21)	0.59 (0.23)	0.66	0.508
IS (confidence)		4.56 (1.79)	4.38 (1.80)	−0.53	0.596
				*t*(63)	*p*
Psychobiological stress		14.09 (35.51)	7.64 (5.05)	−1.06	0.292

*IG* Intervention group, *CG* Control group, *FMI* Freiburg Mindfulness Inventory (range 14–56, higher scores indicate greater mindfulness), *IS* Interoceptive sensibility, *BPQ* Body Perception Questionnaire (range 1–5, higher scores indicate greater interoceptive sensibility), *PSS* Perceived Stress Scale (range 0–16, higher scores indicate greater perceived stress), *BMI* Body mass index (kg/m²), *IAc* interoceptive accuracy (range 0–1, higher scores indicate greater accuracy), *IS *confidence (range 0–10, 0 = total guess/no heartbeat perception, 10 = complete confidence/full perception of heartbeats), *psychobiological stress* cortisol concentrations (pg/mL). Degrees of freedom varied due to missing data

### Changes in mindfulness

The results of the model regarding mindfulness showed a significant fixed effect of the intercept, indicating an estimated mean mindfulness of *β*_*00*_ = 31.433 (*SE* = 0.603; *p* < .001) in the intervention group pre-intervention (T0). There was no significant difference between groups at baseline (*β*_*02*_ = 0.16; SE = 0.857; *p* = .854).

**Fig. 2:**
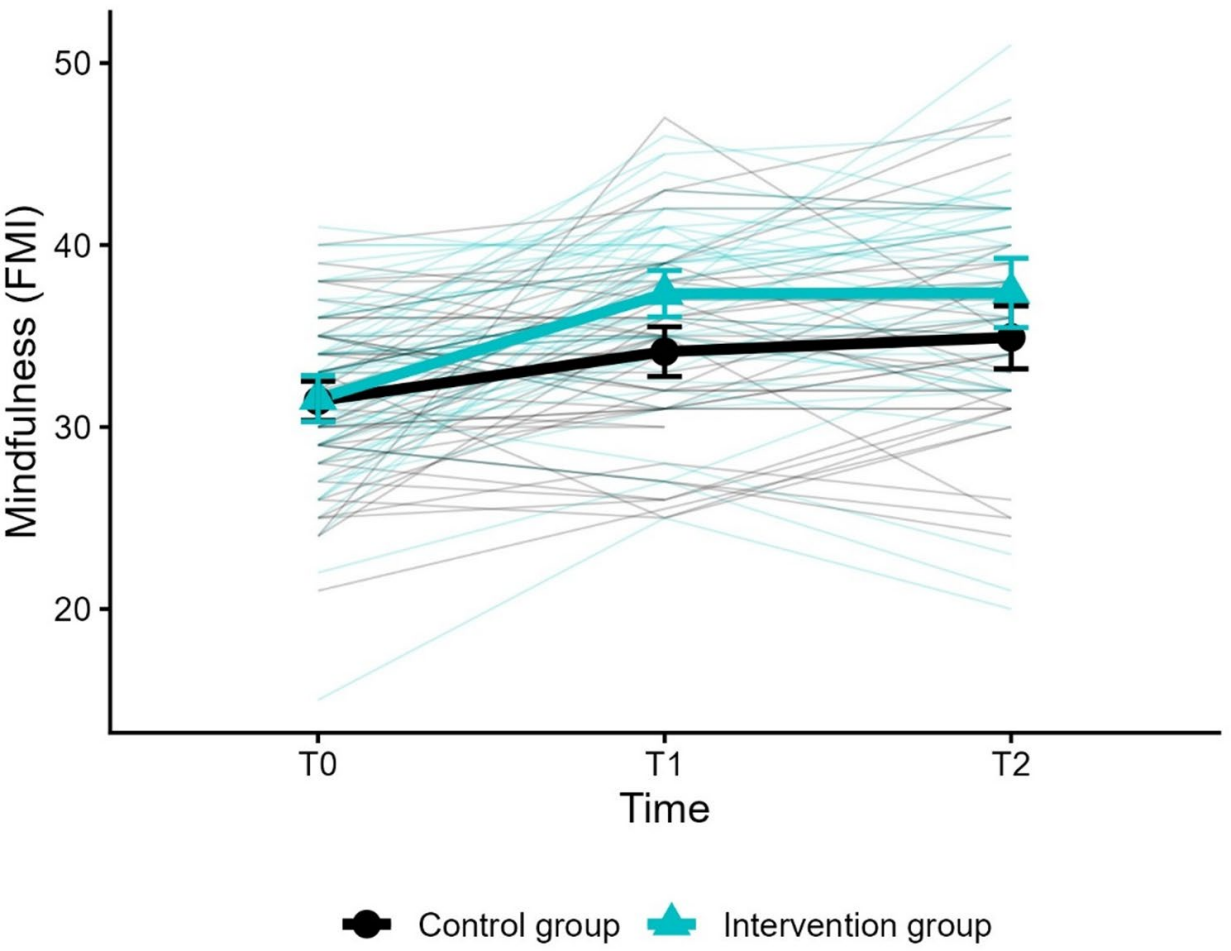
Mean mindfulness scores by group across T0, T1, and T2 with individual trajectories. FMI = Freiburg Mindfulness Inventory (range 14–56, higher scores indicate greater mindfulness); T0 = pre-assessment; T1 = post-assessment (eight weeks after randomisation); T2 = follow-up (six months after randomization). Error bars represent 95% confidence intervals. Thin lines represent individual participant trajectories

The fixed effect of time was significant (*β*_*01*_ = 2.601; *SE* = 0.723; *p* = < 0.001), showing that mindfulness increased over time in both groups. Crucially, the time × group interaction was also significant (*β*_*03*_ = 3.137; *SE* = 1.013; *p* = .002), indicating that the intervention group improved significantly more than the control group, with an average increase of 5.74 points per measurement point compared to 2.60 in the control group (see Table 3). Mean mindfulness scores by group across T0, T1, and T2 with individual trajectories are depicted in Fig. 2.

**Table 3:** Fixed and Random Effects From Multilevel Models Predicting Stress, Mindfulness, and Interoception Outcomes by Time, Group, and Their Interaction

	Mindfulness	Perceived Stress	Psychobiological Stress	Interoceptive Accuracy	Interoceptive sensibility (Confidence)	Interoceptive sensibility (BPQ)
Predictor	β (SE)	β (SE)	β (SE)	β (SE)	β (SE)	β (SE)
Fixed effects						
Intercept	31.443 (0.603)***	6.573 (0.366)***	8.022 (3.394)***	0.600 (0.029)***	4.391 (0.234)***	2.794 (0.090)***
Time	2.601 (0.723)***	0.180 (0.236)	− 0.038 (4.660)	0.002 (0.014)	0.102 (0.119)	− 0.080 (0.055)
Group	0.157 (0.857)	0.639 (0.519)	− 0.999 (4.974)	− 0.033 (0.041)	0.303 (0.332)	0.014 (0.127)
Time × Group	3.137 (1.013)**	− 0.989 (0.329)***	8.413 (7.026)	0.017 (0.020)	0.244 (0.168)	0.057 (0.076)
Random effects, σ² (SD)						
Intercept	8.349 (2.889)	3.693 (1.922)	—	0.038 (0.196)	2.428 (1.558)	0.306 (0.553)
Slope (Time)	—	0.031 (0.175)	—	0.004 (0.065)	0.197 (0.444)	0.019 (0.139)
Residual	13.848 (3.721)	5.156 (2.271)	—	0.010 (0.101)	0.862 (0.929)	—

All models included random intercepts. Random slopes for Time were included unless noted

BPQ  Body Perception Questionnaire, β  unstandardized fixed effect coefficient, SE  standard error, σ² variance component, SD  standard deviation

^a^ Fixed slope model (random slope for Time not included). — indicates parameter not estimated

** *p* < .01. *** *p* < .001

### Changes in perceived stress

**Fig. 3:**
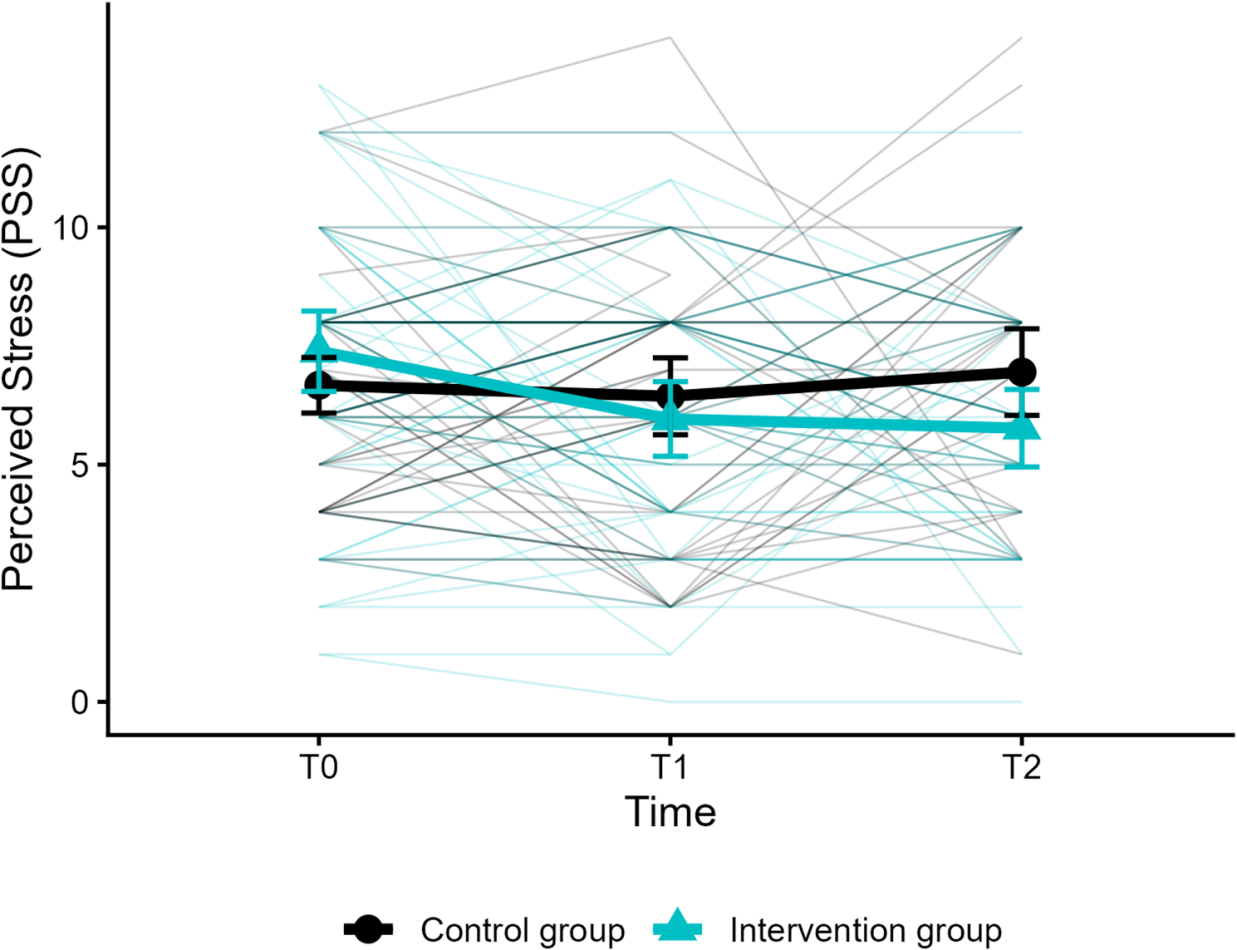
Mean perceived stress scores by group across T0, T1, and T2 with individual trajectories. PSS = Perceived Stress Scale (range 0–16, higher scores indicate greater perceived stress); T0 = pre-assessment; T1 = post-assessment (eight weeks after randomization); T2 = follow-up (six months after randomisation). Error bars represent 95% confidence intervals. Thin lines represent individual participant trajectories

The results of the model concerning perceived stress revealed a significant fixed effect of the intercept, i.e., an estimated mean perceived stress of *β*_*00*_ = 6.573 (*SE* = 0.366; *p* < .001) in the intervention group at T0. The cross-level-interaction of time and group (*β*_*03*_ = − 0.989; *SE* = 0.329; *p* < .001) was significant, indicating that perceived stress decreased significantly more than in the control group. The fixed effects of the level-1-predictor time, the level-2-predictor group, and the random effects were not significant (see Table 3). Mean perceived stress scores by group across T0, T1, and T2 with individual trajectories are depicted in Fig. 3.

### Changes in psychobiological stress

The results of the model predicting psychobiological stress with the predictors time, group, and the interaction time x group revealed a significant fixed effect for the intercept (*β*_*00*_ = 8.022; *SE* = 3.394; *p* = .020). The other fixed effects and the interaction were not significant (see Table 3).

### Changes in interoceptive accuracy

Results show a mean interoceptive accuracy of *β*_*00*_ = 0.60 (*SE* = 0.029; *p* < .001) in the intervention group at T0. Interoceptive accuracy did not significantly change over time (*β*_*01*_ = 0.002; *SE* = 0.014; = 0.896), in the groups (*β*_*02*_ = − 0.033; *SE* = 0.041; = 0.422), neither over time nor the groups (*β*_*03*_ = 0.017; *SE* = 0.020; = 0.414). The non-significant random effects are reported in Table 3.

### Changes in interoceptive sensibility (confidence ratings)

The results of the model predicting interoceptive sensibility (assessed via confidence ratings) revealed only a significant fixed effect of the intercept, indicating an estimated mean interoceptive sensibility of *β*_*00*_ = 4.391 (*SE* = 0.234; *p* < .001) in the intervention group pre-assessment (T0). The other fixed and random effects were not significant (see Table 3).

### Changes in interoceptive sensibility (Body Perception Questionnaire)

Mean interoceptive sensibility (assessed by Body Perception Questionnaire) in the intervention group at T0 was *β*_*00*_ = 2.794 (*SE* = 0.090; *p* < .001). There were neither significant differences over time (*β*_*01*_ = − 0.080; *SE* = 0.055; = 0.146), in the groups (*β*_*02*_ = 0.014; *SE* = 0.127; = 0.911), nor significant changes over both time and group (*β*_*03*_ = 0.057; *SE* = 0.076; *p* = .456). The random effects are reported in Table 3.

### Intervention adherence

Participants in the intervention group completed an average of 7.31 (*SD* = 2.82) out of nine modules (81.2%).

### Exploratory analyses

Table 4 presents the correlations between perceived stress scores and standardised psychobiological stress levels across the three measurement time points of perceived stress (T0, T1, T2) and two measurement points of psychobiological stress. No significant correlations were observed between perceived stress and psychobiological stress at baseline (T0) or post-intervention (T1).

**Table 4:** Correlations Between Perceived Stress Scale (PSS) and Cortisol Levels across T0, T1, and T2

	*N*	1	2	3	4	5	
PSS_T0	121	—					
PSS_T1	103	0.65	—				
PSS_T2	84	0.64	0.27	—			
Cortisol_T0	52	0.49	−0.14	−0.21	—		
Cortisol_T1	59	−0.45	−0.40	−0.08	0.36	—	

Cortisol values refer to standardized cortisol amounts per hair sample

*PSS* Perceived Stress Scale, *T0* Pre-assessment, *T1* Post-assessment, *T2* follow-up assessment

*p < .05

## Discussion

The aim of the present study was to investigate the effects of an eight-week online mindfulness-based intervention on mindfulness, interoceptive abilities, perceived, and psychobiological stress in students from pre- to post- assessment and to a six-month follow-up.

### Effects on mindfulness

In line with our first hypothesis, mindfulness increased significantly due to the online MBI, with results revealing a significant increase from pre- to follow-up assessment and a significantly stronger increase compared to the control group. This finding aligns with the substantial body of evidence demonstrating that structured MBIs, including online formats, effectively enhance mindfulness skills [31, 39, 40, 44, 46, 47]. Of particular relevance is the comparison with other StudiCare Mindfulness trials [41, 48] which evaluated previous versions of the intervention for university students. While Küchler et al. [48] found significant within- and between-group improvements in mindfulness immediately post-intervention in a guided version of the online MBI without follow-up assessment, Küchler et al. [41] extended these findings by reporting significant between-group effects – also at 6-month follow-up – for an unguided version of the online MBI compared to a guided-on-demand format. Our study further contributes to this line of research by demonstrating sustained mindfulness improvements at follow-up in a guided online MBI, suggesting that such interventions can foster enduring mindfulness skill acquisition over time.

This finding demonstrates the effectiveness of the online MBI in enhancing mindfulness, which substantiates the appropriateness of the intervention content. The results support the proposed mechanisms of MBIs, suggesting that enhanced attentional control and improved emotion regulation may have contributed to the improvements in mindfulness [38]. Comparably to the interpretation of the results by Küchler et al. [48], the effect might also be partly attributable to the guidance of the intervention. Furthermore, the effect may have been further supported by the high intervention adherence, with participants in the intervention group completing an average of 7.31 out of 9 modules (7 core modules + 2 booster modules), representing an overall mean intervention adherence of 81.2%. In contrast, previous studies based on the StudiCare Mindfulness intervention reported lower adherence rates of 3.57 modules (70% [48]), and 2.51 [41]. The high intervention adherence observed supports the feasibility of guided, web-based mindfulness training for university populations. In particular, the regular in-person contact during laboratory assessment sessions may have further contributed to participant retention.

It should be noted that participants were selected based on low to moderate baseline mindfulness levels (FMI < 37), targeting individuals with the greatest potential need for mindfulness training, consistent with other online MBI trials using the same cutoff [41, 48]. Although the randomised controlled design mitigates regression to the mean, this restriction may have increased the likelihood of observing larger pre-post improvements. This is supported by a recent meta-analysis of 177 RCTs [101] which found that lower baseline trait mindfulness was associated with larger improvements following MBIs, and by Kiken et al. (102) who showed that individuals’ baselines mindfulness shape their response to mindfulness training. The present effects may therefore not generalize to student populations with higher baseline mindfulness, and future studies should systematically test baseline mindfulness as a moderator of intervention outcomes.

### Effects on perceived and psychobiological stress

Perceived stress decreased due to the online MBI from pre- to follow-up intervention compared to the control group, which is in line with our hypothesis and previous findings [31, 39–41, 43, 44, 46, 47]. The present study demonstrates that the intervention was effective in reducing perceived stress in the intervention group with participants showing improvements from baseline to follow-up, compared to the control group. This finding aligns with a recent systematic review and meta-analysis [29] which reported small to medium effect sizes for stress reduction as mostly assessed via the Perceived Stress Scale (10-item version, PSS-10), when online MBIs were compared to non-active controls at post-intervention. However, Alrashdi et al.[29] noted that these effects were not sustained when online MBIs were compared to active controls or at follow-up assessments. Significantly, our findings show a lasting effect at follow-up intervention, compared to a waitlist control group.

In contrast to the significant reductions observed in perceived stress, no intervention effects were found for psychobiological stress as measured by hair cortisol. Furthermore, mainly no significant correlations between perceived and psychobiological stress parameters were found, besides from negative associations between psychobiological stress measured at follow-up and perceived stress measured at baseline and follow-up.

Our findings diverge from those reported by Schultchen et al. [55] who examined an 8-week body scan intervention and found significant improvements in biological stress markers, with decreased cortisol and cortisol/DHEA in the intervention group compared to an audio book control group. Notably, Schultchen et al. [55] also observed that perceived chronic stress as assessed via the Trier Inventory of Chronic Stress [103] decreased in both the intervention and the control group, and critically, they found no correlation between psychobiological and psychological stress markers. Similarly, Gherardi-Donato et al. [54] conducted a randomised clinical trial with university workers and reported that psychobiological stress as assessed via hair cortisol, and perceived stress were significantly reduced after an eight-week mindfulness intervention compared to the control group.

However, our lacking effects on psychobiological stress are not entirely inconsistent with the broader literature. In particular, our results are in line with the findings by Jong et al. [56] and Repo et al. [57], showing no effects of online MBI [4 weeks, 8 weeks] on psychobiological stress (hair cortisol) in student samples. Finally, a systematic review and meta-analysis [104] examining the effects of MBI on biomarkers of inflammation and stress in healthy, stressed, and at-risk populations found only small effects [Hedge’s *g* = −0.15 for pre-post changes].

The absence of significant effects on hair cortisol can be interpreted in several ways. MBI often have a stronger impact on how individuals perceive and interpret stressful experiences than on physiological parameters, which typically change more gradually [104]. Moreover, the PSS-4 and hair cortisol reflect different temporal perspectives, with perceived stress covering the past four weeks and hair cortisol representing cumulative cortisol secretion over approximately eight weeks. As a result, short-term psychological changes may not be captured in this long-term biological marker. In addition, chronic or prolonged stress can lead to a downregulation of the hypothalamic-pituitary-adrenal (HPA) axis, resulting in low or stable cortisol levels despite high perceived stress. Methodological factors such as individual differences in hair characteristics, washing frequency, or the relatively small sample size may have further limited the detection of small effects. Overall, these findings suggest that the intervention primarily affected perceived stress, whereas physiological adaptations may be more subtle or delayed. This dissociation between perceived and psychobiological stress is consistent with theoretical accounts suggesting that mindfulness primarily operates on the appraisal and regulatory level of stress processing rather than directly on HPA axis activity [104].

#### Effects on interoceptive abilities

Contrary to the second hypothesis, interoceptive accuracy (IAc) did not increase following the online MBI. To the authors’ knowledge, this is the first study investigating the effects of an entirely 8-week-online MBI on interoceptive abilities. Our lacking effects on interoceptive accuracy align with Parkin et al. [74], who reported no effect of an 8-week MBI on IAc. At the same time, other studies did find improvements in interoceptive abilities due to diverse in-person MBIs over different time periods [72, 105, 106].

Within predictive coding frameworks [107, 108], IAc indexes lower-level interoceptive signal detection, i.e., the match between ascending visceral afferences and the brain’s interoceptive predictions [107]. Improving IAc would require increasing the precision weighting of interoceptive prediction errors through sustained, focused bodily attention [71]. Accordingly, interventions with a concentrated interoceptive focus, such as body scan protocols, have demonstrated improvements in IAc [72, 105, 109]. In contrast, the MBI of the present study contained various contents including mindfulness-based, body-focused contents, and stress-management-based elements. Therefore, the contents differ in their types of tasks and modules, and especially, some of the described interventions’ contents not entirely focusing on the body as compared to, e.g., the body scan interventions. Different mindful exercises might lead to different bodily experiences or processes concerning interoception [60, 70, 110].

Concerning IS, no effect was found due to the online MBI. In line with our results, the study by Schillings et al. [76] could not show any effects of a 3-week online MBI guided by a chatbot on IS. In contrast, several previous studies showed improvements in IS in the context of in-person MBI [74, 109, 111–113].

Within the predictive coding framework, IS reflects higher-order metacognitive priors about one’s own interoceptive accuracy [107, 108]. Such beliefs tend to be relatively stable, as their revision requires strong, repeated discrepancies between expected and actual interoceptive performance. An 8-week online MBI like in the present study not specifically designed to generate such metacognitive confrontation may be insufficient to shift these consolidated models. This is consistent with the finding that a chatbot-based MBI targeting several health-related constructs did not lead to improvements in IS [76].

Considering the results of IAc and IS, besides differences in the interventions’ lengths between the described studies person MBI [74, 76, 109, 111–113], additionally, the intensity of the intervention, e.g., how often and in which time intervals participants conduct the intervention in everyday-life, might be a crucial factor. Moreover, significantly, the assessment of interoceptive abilities was diverse. Lastly, the present sample of our study was not explicitly characterised by low interoceptive abilities. Future studies should also investigate samples exhibiting low interoceptive abilities such as chronically stressed individuals [68] or individuals with mental disorders such as eating disorders, e.g., anorexia nervosa [73, 114].

#### Limitations and future research

Several limitations should be acknowledged. First, the sample was predominantly female, which may limit the generalizability of findings. Second, as discussed, substantial dropout occurred at the follow-up assessment and a considerable number of hair cortisol samples had to be excluded from analyses, further reducing the available data for psychobiological stress assessment. Third, dehydroepiandrosterone (DHEA), an endogenous steroid hormone that counteracts cortisol effects [115] could not be analyzed in this study. The cortisol/DHEA ratio is a measure of endocrine imbalance of the HPA axis and an indicator of mental stress in healthy humans [55, 115, 116]. Future studies would benefit from a multi-system biomarker approach, incorporating measures of HPA axis functioning (cortisol and DHEA), autonomic nervous system activity (heart rate variability, salivary alpha-amylase), and inflammatory markers (e.g., C-reactive protein, interleukin-6) [117, 118]. Such an integrative approach could clarify whether effects of online MBI manifest differentially across physiological systems and time courses. Moreover, the assessment of perceived stress should be complemented by the assessment of chronic stress, e.g., via the Trier Inventory of Chronic Stress [103]. Fourth, primary analyses were not adjusted for covariates, as randomisation successfully balanced baseline characteristics across groups. Nevertheless, future studies may consider covariate-adjusted sensitivity analyses. In addition, reasons for participant dropout were not systematically assessed. One known contributing factor, however, was the COVID-19 pandemic, as restrictions during the data collection period meant that some on-site assessment appointments could not be conducted. Future studies should collect this information systematically to better characterise attrition patterns.

Furthermore, IAc was assessed using the heartbeat counting task [82], a widely used measure of cardiac interoception. While its validity has been discussed in the literature, with some authors pointing to potential influences of prior knowledge, beliefs about heart rate, or guessing strategies [119–122], it remains a commonly applied and informative tool in interoception research. To minimize such influences, studies recommend providing clear instructions emphasizing that participants should only report heartbeats they actually perceive [119], which was implemented in the present study. In addition, recent work has proposed complementary approaches, including alternative cardiac tasks e.g. [123, 124], and multimodal assessment methods spanning different interoceptive domains e.g., [125, 126]. Future research may benefit from combining such measures to obtain a more comprehensive assessment of interoceptive processes.

## Conclusions

The guided online MBI effectively enhanced mindfulness and reduced perceived stress in university students, with sustained effects at 6-month follow-up. However, the intervention did not significantly affect psychobiological stress markers or interoceptive processing. These findings suggest that online MBIs primarily target psychological rather than psychobiological stress dimensions, and highlight the need for longer intervention periods or multi-system assessment approaches to detect potential physiological changes. At the same time, the absence of effects on psychophysiological measures should be interpreted with caution, as these outcomes may have been subject to additional uncontrolled sources of variability. The high intervention adherence observed supports the feasibility of guided, web-based mindfulness training such as StudiCare Mindfulness for university populations.

## Data Availability

The data that support the findings of this study are stored on password-protected servers with restricted access. Anonymised data are available from the corresponding author upon reasonable request, subject to data protection agreements and ethical approval.

## Abbreviations

MBI: Mindfulness-based intervention
IMI: Internet- and mobile-based intervention
FMI: Freiburg Mindfulness Inventory
PSS-4: Perceived Stress Scale (4-item short form)
IAc: Interoceptive accuracy
IS: Interoceptive sensibility
BPQ: Body Perception Questionnaire
HPA: Hypothalamic-pituitary-adrenal axis
MBSR: Mindfulness-Based Stress Reduction
ACT: Acceptance and Commitment Therapy
DHEA: Dehydroepiandrosterone
